# The perceived impact of family medicine leadership training on practice: A qualitative study

**DOI:** 10.4102/phcfm.v17i1.4954

**Published:** 2025-08-27

**Authors:** Samantha Dladla, Klaus B. von Pressentin, Tasleem Ras

**Affiliations:** 1 Division of Family Medicine, Department of Family, Community and Emergency Care, Faculty of Health Sciences, University of Cape Town, Cape Town, South Africa

**Keywords:** leadership, clinical governance, qualitative evaluation, family medicine, fit for purpose

## Abstract

**Background:**

Family physicians (FPs) play a crucial role in clinical governance within South Africa’s District Health System, necessitating strong leadership skills.

**Aim:**

To understand how the postgraduate Leadership and Governance module at the University of Cape Town (UCT) helped prepare qualified FPs working in the Western Cape province public sector for their leadership role.

**Setting:**

The UCT offers a 4-month Leadership and Governance module as part of its 4-year Master of Medicine in Family Medicine programme, aiming to prepare registrars for leadership roles.

**Methods:**

An exploratory qualitative study design was used. A total of 10 UCT alumni working in senior public sector roles were purposively sampled for online semi-structured interviews. Interviews were recorded, transcribed and analysed using the framework method. Data were coded deductively into themes, with new themes created for cohesive uncategorised data.

**Results:**

Findings revealed that FPs shared similar early experiences as clinical leaders and faced a transitional phase after completing their registrarship. While key leadership qualities aligned with existing literature, participants emphasised the importance of context-specific training and the value of community practice resources.

**Conclusion:**

The module itself was not considered particularly helpful in preparing FPs for real-world leadership and governance challenges.

**Contribution:**

The study highlights gaps between theoretical training and practical leadership demands, indicating that the module must better address the realities faced by newly qualified FPs. This research contributes to understanding the limitations of current leadership training and underscores the need for more practical, contextually relevant education for FPs in leadership roles.

## Introduction

### Background

Family medicine (FM) has been recognised as an established specialist discipline by the Health Professions Council of South Africa (HPCSA) since 2007, with about two decades of various forms of postgraduate training preceding this date. The formal recognition of FM as a specialised discipline ushered in the 4-year registrar programme across nine universities in the country.^1^ In Africa and South Africa, many authors have discussed the changing environment within primary and district health care as well as the importance of effective leadership in the form of family physicians (FPs).^2,3,4,5,6^ Ilori et al. highlight FPs’ role in improving the quality of care and outcomes in Nigeria, specifically regarding equity of care.^6^

South African (SA) training programmes adopted, as a core component of their curriculum, the teachings of Ian McWhinney, a Canadian professor of FM, who outlined the foundational principles of the discipline.^7^ He emphasised nine principles, all equally important to FM and primary care. One particular principle, namely resource management, was described by McWhinney, highlighting how FPs must utilise their skills as first-contact physicians to manage resources. This becomes especially poignant in certain parts of the world where resources are extremely limited.

Within South Africa, many challenges relating to persisting inequalities in the healthcare system stem from the political past. Even though progress is being made towards universal health coverage (UHC) outlined in policy,^2^ many communities still face significant difficulties in accessing and receiving health care services because of financial, structural and governance issues. A more considerable emphasis has been placed on decentralising health care within South Africa and using teams in communities to enhance access to both primary and appropriate specialist care.^2^ With this reinforced mandate to enhance access to equitable primary health care for SAs, ensuring better governance and management of human and other resources is imperative, reflecting a function of effective leadership. In the SA context, FM training programmes provide leadership and governance training to registrars, preparing them for their future roles as FPs.^8,9^

In the SA context, Mash continues to explore the latest contributions of FPs to health care and reports that the their role and contribution are still primarily clinical.^10^ Family physicians made a relatively minor contribution to governance in SA health care specifically, and this could be for several reasons.^10^ Much of this stems from the organisational and structural hierarchies within the SA health care system, within which the FPs must assert themselves. These findings suggest that the FPs’ ability to lead and govern is not yet fully recognised. There remains considerable ambivalence regarding the leadership qualities of the FP, as articulated by prominent leaders in government and policy development.^3,4^ The questions that arise are: What is a leader? What is a leader within the health care sector? What attributes constitute an effective leader?

A systematic review of leadership within the healthcare system done by Mukwankungu et al. explains the numerous challenges clinicians encounter within the healthcare and managerial spheres.^11^ They highlight that many health practitioners lack not only the necessary skills but also the confidence to undertake leadership roles, which compromises the administrative performance of the health system. They also outline the specific training leaders would need to bolster their confidence and skills, as well as the theories they would have to familiarise themselves with. They report that clinicians are not designated for this training but are often asked to step up and manage units and facilities. It is also noted that leadership training for doctors and nurses would differ. Despite courses and managerial programmes being available, their efficacy is yet to be proven.^11^ The clinician may also prefer to concentrate more on the clinical aspect of their role, as this is their primary area of expertise, rather than the managerial aspects.

Gilson and Daire outline three key leadership abilities that can transform health care:^12^

- The ability to use the extensive variety of information and data in decision-making to identify constraints within operations.
- Involve people in decision-making rather than imposing.
- Develop strong relationships with higher levels of political support and other resourceful parties outside the system.

The above-stated skills may seem to be in addition to clinical training and have been noted to be outside the scope of undergraduate programmes, but they are within the scope of a trained FP.

The idea of a lack of confidence regarding leadership was reiterated by Gallagher et al., who conducted a cross-sectional quantitative survey with junior registrars in Canada.^13^ This survey reaffirmed that FPs see themselves as leaders after training, but even registrars felt that they needed more specialised training concerning leadership and governance. Their curriculum only deals with basic leadership skills and knowledge. They suggested more in-depth teaching on other advanced concepts such as administration, coalitions and system transformation.^13^ These advanced concepts align with Gilson and Daire’s key abilities for leadership, which could suggest an interesting framework for thinking about a leadership curriculum for clinicians.

The proposed roles and expectations of the FP within the SA context are explored further in the latest position paper of the South African Academy of Family Physicians (SAAFP).^9,10^ This paper describes the FPs’ scope of practice and training and how they are trained in leadership across all their roles. However, it is noted that FPs are not trained primarily to be managers.^10^ Many policies discuss the need for better coordinated decentralised care for communities, better governance and resource management, and strong leadership.^2,3,4,5,6^ Family physicians are ideally trained for primary and district-level care, and cannot be effectively utilised in tertiary settings. Their extensive training and skill set position them as key players in the district health system when implementing policy and rectifying systems as needs arise within each community.^10^ As South Africa develops and seeks to bridge the gap of equality within the nation through essential service delivery and policy implementation to address historical injustices, the leadership role of FPs within primary care has become increasingly significant.

The national learning outcomes (NLOs) within the national portfolio of learning for FM training, which forms the basis of the Master of Medicine (MMed) programme to ensure uniformity across South Africa, have a specific outcome dedicated to leadership and governance.^8,14^ This outcome outlines the skills and attributes that must be learned and practised throughout the 4-year training programme. While these attributes may differ from those listed by Gilson and Daire, they intersect and are possibly explored more comprehensively within the FM programme. Mash et al. discussed, in the upgraded programmatic learning outcomes, how the initial learning outcomes emerged in 2012 and the significance of regularly revising these outcomes, guided by the needs of our health system.^8^ The discipline agreed on the new guidelines published in 2021 were based on the actual competencies observed and utilised by FPs over the preceding decade.^8^ Mash also suggests the need to potentially expand and enhance the curriculum, considering the contribution of FPs, to ensure that the speciality is effectively utilised within the healthcare system.^10^

The Leadership and Governance (L&G) module offered during the registrar training programme at the University of Cape Town (UCT) was revised in 2022 to align with the latest NLO published, as well as taking into consideration points raised in both publications by Mash et al.^6,8^ The course outline before 2021 was oriented towards clinical service delivery, with key topics covered over seven modules. The main topics were discovering individual personality profiles, leadership within the district health system, understanding corporate governance and looking at specific clinical governance activities. These key topics align with the latest position paper by Mash et al., suggesting again that the contribution from qualified FPs remains largely clinical.^7,8^

The key components of the updated 2022 L&G module are now presented over an expanded 12-session module and include sessions involving a group coaching approach dedicated to leadership development, sessions on clinical and corporate governance content, a leadership or personality style assessment measured by the Enneagram^15,16^ and positioning all the content sessions within a reflection on context to assist with transferring learning into practice. The key changes in the latest module highlight points relevant to South Africa’s evolving healthcare system and mirror some of the leadership qualities mentioned by Gilson and Daire.^12^ The improvements were also informed by previous graduates’ reflections, which proved valuable in helping to shape the dynamic process of curricular reform linked to this evolving module.

The SAAFP has started a new initiative in 2021, the Next5 special interest group, to help newly qualified FPs develop in their roles and careers through mentoring and certain activities as determined by member needs.^17^ The SAAFP surveyed members to identify learning gaps that could be addressed within this special interest group. The selected activities highlighted themes such as communication and marketing, graduation and registration, and mentoring and networking. There is no mention of specific managerial topics or the reinforcement of leadership and governance qualities. This may indicate an unidentified need; however, it is also important to note that only registered SAAFP members could contribute to the survey. The number of registered members mentioned in the same article, although increasing, remains low compared to the actual number graduates.^17^

Within South Africa, a few research papers have been written attempting to understand and evaluate different teaching interventions within FM. Many of these articles have been qualitative studies exploring experiences of various aspects of the FM post-graduate curriculum.^18,19,20^ None of the these articles have addressed specific teaching interventions around a particular L&G module in the country or looked at the experiences of the newly qualified FPs. Britz et al. have discussed the need for an assessment framework related to clinical assessments and raised valid points about the challenges around maintaining assessment quality. They mentioned that assessments are multi-dimensional and that no assessment is perfect. This is particularly interesting in the setting of clinical medicine, but it also helps validate how even more challenging assessing a softer, less tangible skill like leadership and governance would be in the setting of a structured curriculum.^21^

Although the need for clinical leadership in primary and district care services has been addressed within the curriculum, but more needs to be known about the effectiveness of formal leadership training. This represents a significant gap in the educational literature in relating training programmes to clinical platforms.

The study aimed to understand if and how the previous postgraduate L&G training module in the MMed curriculum at UCT helped prepare newly qualified FPs for the Western Cape province of South Africa’s public sector. The main objectives were:

- To describe participants’ experiences of the L&G module concerning their work.
- To identify learnings from the L&G module perceived as useful by participants.
- To describe competencies identified by workplace experience not covered in the curriculum.
- To identify additional resources participants used to enhance their leadership competence once practising.
- To identify perceptions of effective leadership attributes needed on the district clinical platform.

## Research methods and design

### Study design

A narrative analysis using an interpretive and exploratory strategy was adopted in this qualitative research design, as described by Mabuza et al.^22^

### Setting

This study focused on FPs practising at the Department of Health (DOH) facilities. It primarily examined their educational exposure to the UCT module and tracked those FPs specifically based within the Western Cape province.

These facilities within the province can be divided into rural or metro sites. The Cape Metro health district has eight legislated sub-districts serving over 4.1 million people, according to the latest population statistics from 2018 to 2019. There are 152 primary health care facilities and 8 district hospitals. There are also tuberculosis (TB) hospitals, but they support the broader Western Cape in delivering TB inpatient services.^23^ The rural sites are defined as all the municipalities outside the Cape Metro health district, which complied with the definition of ‘rural’ as being 1 h or more by road transport or 80 km from a referral centre.^24^ The range of services covered at a typical district health facility includes:^25^

- Child health.
- Emergency medical services.
- Family planning.
- Forensic pathology services.
- Women’s health.
- Tuberculosis and human immunodeficiency virus (HIV)/acquired immunodeficiency syndrome (AIDS).
- Men’s health.
- Psychiatric services.
- Home-based care.

### Study population and sampling strategy

Participants were purposively sampled with specific inclusion and exclusion criteria, as shown in Table 1. A total of 13 FPs were invited to participate; however, data saturation determined the sample size. Participants were contacted via email requesting their voluntary participation in the study from an alumni list compiled by the Division of Family Medicine. Because of various challenges, including the availability of consultant posts after graduation and the response rate of participants to this study, specific criteria regarding the years following exposure to the MMed programme had to be extended to assist in recruiting participants.

**TABLE 1:** Sampling strategy.

Inclusion	Exclusion
FPs who have completed the UCT four-year MMed training programme in the last 3–7 years with or without completion of their research component.	FPs not trained in South Africa or not exposed to the UCT L&G module during their 4 years of MMed training. FPs trained at any other institution.
FPs noted to be in formal leadership positions or consultant FP posts.	-
FPs working in the public sector in WC.	-

FPs, family physicians; WC, Western Cape; UCT, University of Cape Town; L&G, leadership and governance.

### Data collection

The research team consisted of the primary researcher and her two supervisors.

Data were collected through semi-structured interviews with an interview guide and an initial demographics portion (Appendix 1).^26^ The interview guide was developed in alignment with the stated objectives and submitted with the original protocol. It was piloted on two senior FM registrars and amended accordingly.

The primary researcher conducted the interviews at the participants’ convenience. All interviews took place online using the Zoom meeting platform. Interviews were conducted in English and recorded using the same platform. Professional transcription services manually transcribed the audio recordings. Before analysing the data, the transcriptions were checked for accuracy, and mistakes in transcription were corrected against the original recording.

#### Data analysis

The researchers familiarised themselves extensively with the data, listening to the audio recordings and reading the transcripts. This was done repeatedly and in a phased approach, as the transcriptions took time to generate following each interview.

The data were analysed deductively using the framework approach.^27^ The framework was constructed by reviewing the literature on clinical leadership models, FM (globally and locally) and an initial data analysis.

Categories were generated from this process that included two initial themes: ‘key leadership qualities required’ and ‘fit-for-purpose training’. During coding, two further categories emerged, reported next, providing a framework with four categories, later termed themes because they contained data that provided a cohesive narrative. Initial codes were extracted from the data and categorised within the framework. Sub-themes and themes emerged from these codes. The primary researcher performed coding, and the rest of the team assisted with quality control as described next.

#### Quality control

Adhering to the Lincoln and Guba, trustworthiness criteria^22,28^ assured data quality. Prolonged engagement with the data and researcher triangulation during the analysis phase using field notes and interview transcriptions ensured credibility. This also attempted to reduce observer bias.

Member checking, also known as respondent validation, was performed by presenting the study’s preliminary findings at a group forum attended by participants. The presentation and themes resonated well with the group, and no additional data were generated, indicating that data saturation had been achieved in the semi-structured interviews.

It is difficult to completely exclude selection bias in this study, as the participants were purposely sampled from a list compiled by the Division of Family Medicine. All participants who were contacted worked in the government sector per the inclusion criteria, but the number of participants who consented was out of the researcher’s control.

Data were collected over an extended period using thick descriptions with a clear audit trail to achieve transferability. The interview guides were piloted, interviews were audio recorded and transcribed to ensure accurate records, and the analysis process was supervised following training in qualitative research analysis. This also helps ensure dependability.

Regarding reflexivity, the main researcher, currently in her final years of residency, has extensive experience in South Africa’s primary and district health care systems. She acknowledges that intuitive knowledge helped inform her research question and that personal bias exists as she is embedded in the training programme. However, attempts were made to minimise this bias through conversations with the supervisors, personal reflections and the above-stated steps to help ensure the trustworthiness of this study. She approached the analysis methodically and engaged with only the data provided to manage subjectivity.

### Ethical considerations

This study was guided by the Declaration of Helsinki on medical research involving human subjects.^29^ Ethical approval to conduct this study was obtained from the University of Cape Town Faculty of Health Sciences Human Research Ethics Committee (No. 342/2003), and written informed consent was obtained from all participants before data collection.

## Results

### Demographics

In all 13 FPs were invited to participate in the study. Two FPs did not respond despite several communication attempts, and one FP declined to participate in the study as she felt she was not in a typical FP role. A total of 10 participants were interviewed, of which 7 were male. The youngest participant was 34 years old, and the oldest was 53 years old. The years of graduation vary, with 50% of the candidates graduating between 2018 and 2022. Seven participants were in FP consultant posts, one was a clinical manager, and two were in medical officer (non-consultant) posts because of a scarcity of FP posts.^30^

### Themes

The data were organised into sub-themes and themes (Table 2). Saturation was reached by the sixth interview. However, data collection continued to ensure that all 10 participants were interviewed.

**TABLE 2:** Themes and sub-themes.

Theme	Sub-theme
1. The *early experiences* of FPs as clinical leaders	A challenging start
	Realisation of the broad scope of practice
	Various job availability at the time of qualifying
2. Self-identified *key leadership qualities* required from FPs	Communication and transparency
	Absence of traditional hierarchy
	Relationship building, teamwork and skills development
3. Fit-for-purpose training	Concern about the practicalities of the actual job description not being adequately covered
	Knowledge gaps specific to administrative skills, governance roles and responsibilities
4. *Resources used by FPs* to overcome perceived shortcomings of formal training	Making use of a community of practice
	Governmental circulars and courses

FPs, family physicians.

### The early experiences of family physicians as clinical leaders

#### A challenging start

During this study, it became evident that despite the differences in years of work experience between the participants, most still found their time as clinical leaders a positive experience. Many shared the sentiment of a challenging start, but usually, with time and experience, there was an improvement in both skill set and confidence within their role. One participant stated:

‘It’s been rewarding but an interesting rollercoaster. I think that first few years were really tough.’ (1.28, 43 year, F)

Many expressed that even after completing their formal training, there were still many knowledge gaps regarding their leadership roles, many of which were filled with on-the-job training. One participant reported:

‘It was very difficult in the beginning, like finding my feet, and there’s a lot of things that are not taught that you kind of have to learn on the job.’ (4.41, 39 year, F)

#### Realisation of the broad scope of practice

Upon reflection on their journeys to this point, many participants shared an overwhelming realisation and acceptance that their potential scope of practice is so vast. ‘So … there is much that is involved in the role’ (3.45, 47 year, M). Some participants embraced that challenge and utilised all their skills daily within their sphere of influence: ‘Everything I have studied during registrarship really comes into fruition when you are actually in the role, like in the role because you do everything’ (10.86, 39 year, F). While others still grapple with their roles and responsibilities, which are no longer primarily clinical: ‘Administrative things that leadership people in the state sector should know about and that I found that I didn’t know much about’ (8. 126, 34 year, M). Senior participants expressed a more mature view towards previous expectations of a FP, stating that during their training, FPs were not considered to function as managers and that it was impossible to cover everything: ‘We were brand new. So, look in, in, in all fairness, I don’t think they knew what they were teaching us in the beginning anyway’ (7.125, 47 year, M).

#### Various job availability at the time of qualifying

A driver that influences where FPs work, their work experience and subsequent roles as leaders is job availability at the time of graduating. Family physicians in all year groups have expressed different experiences of this with one of the more senior candidates stating: ‘… there were jobs available because we were the first lot to finish’ (7.74, 47 year, M). Conversely, many of the junior FPs have had to find medical officer (non-specialist) posts while waiting for FP or clinical manager posts to open up: ‘So I’m the medical officer … family physician … working as (in) a medical officer post’ (6.24, 47 year, M). One participant, post-graduation, had to find work overseas because of job scarcity and found himself now with a different challenge being:

‘I’m a senior doctor in the cruise industry … So, I work in the maritime healthcare space. I am a doctor at sea, but I still do locum work when I’m on holiday, in Emergency Rooms (ERs) in Cape Town … my biggest challenge now is that I haven’t worked in a family physician post in South Africa.’ (2.38, 47 year, M)

### Self-identified key leadership qualities required from family physicians

#### Communication and transparency

Participants were able to reflect on their years of lived experience and draw on some of the key learning points about the leadership skills required: knowing and building up your team, transparency and communication. Participants were able to express a sense of self-awareness and awareness of their teams, and could acknowledge points that their teams valued. One of these was sharing information and transparency regarding the workings of the facility and staff members: ‘So … whether that’s integrity or transparency, but transparency also in this is how the system works’ (1.371, 43 year, F).

#### Absence of traditional hierarchy

By using the bottom-up approach, they were able to create a culture which aims to decrease the old traditional hierarchies often present within the health sector and use what one participant describes as:

‘I also think that bottoms-up approach … so … I think also the way that you need to be very open to innovation and change. Okay … [*s*]o, it’s not meant to be a hierarchy.’ (4.343, 39 year, F)

This practice aids in the FP getting to know their team members, more than just on a skills level. They can draw on team members’ strengths as well as navigate and eventually work on their weaknesses, therefore building and strengthening the team up as a unit, with one participant reflecting: ‘You need to know what the other people are doing and how you can lean on them, and how they can lean on you’ (7.725, 47 year, M), and another participant similarly stating:

‘You need to have, have an understanding of the system. You need, and I think that’s why I think family physicians are, are, are quite good because we don’t just look at a single speciality. You need to, you need to have a little bit of a bird’s eye view of what’s going on and be respectful and understanding of, you know, what’s, what the nursing side is, what the, you know, the support services side because it’s not just the doctor that makes a hospital run.’ (7.260, 47 year, M)

#### Relationship building, teamwork and skills development

This emphasis on relationship building and respect for their team members is important because, as people, life continues to happen outside of the workspace. The importance of preserving the work-life balance among the team helps build resilience and creates the culture at work where people want to do more because they feel valued as team members. This sensitivity is reflected by one participant stating:

‘… on the team that we have and having some small things, that sort of build resilience, but also its protective of, of people not burning out … Is key, and I mean that, that goes from like having a, a coffee club at work, having specific social events and a lot of, you know, sort of not building a culture of people working for each other … And that’s sometimes challenging, but I think we are working on it.’ (8.279, 34 year, M)

They were also able to make decisions using all available data and resources and, when appropriate, involve the team in this process, as reflected by one of the participant: ‘The other thing is whenever a decision is being taken, it is important to seek the opinion, the view of the team as a whole’ (3.183, 47 year, M). This teamwork in decision-making and policy implementation solidifies the sub-theme of doing away with traditional hierarchies, emphasising the importance of working well within and between teams, as one participant indicated:

‘… so teamwork is, is also a very important attribute that you must, must, must have because you need to be able to work well with the other heads of departments and also work well with the staff and I feel if you don’t have that teamwork, then it causes very poor work ethic in the workplace.’ (4.351, 39 year, F)

Another quality of being a leader and a core value within FM is to upskill and capacitate the team. One participant reported that this is an enjoyable part of the job:

‘Because like yes, we need to teach a lot, but we need to also be open to learning, like continuously learning. And then I think that I also … I love teaching. So, like teaching and training the staff to do the right thing.’ (10.208, 39 year, F)

Investing in staff development, whether through in-service training or specific training, is highly valued, suggesting that the participants understand the needs of their team: ‘I think being a trainer, so like, I think staff like to be upskilled. Staff like to be upskilled all the time’ (4.357, 39 year, F).

### Fit-for-purpose training

This is when training programmes ensure that local needs and context are being met with their curriculum, which includes both theoretical and practical knowledge and aims to better equip the graduate for their future role and responsibilities.^31^

#### Concern about the practicalities of the actual job description not adequately covered

Some participants expressed concerns that some of the practicalities of the job were not addressed in their formal training. Participants identified particular gaps linked to the knowledge of systems and human resource processes that they needed to be effective. Comparing their preconceived ideas about leadership and the actual experience thereof, one participant reflected that: ‘I found that it was very different once we were in the real sort of life doing it’ (8.85, 34 year, M).

For those participants who found the module useful, their perception of the module’s value was heavily centred around personality or leadership style testing, which was a prominent feature throughout the various iterations of the module. Participants were able to think critically about what kind of leader they could be and how others may perceive their styles. Participants benefited from this self-reflection based on an established psychometric model with one of them stating:

‘I really enjoyed the theory of what, of sort of stimulating what your own leadership style might be and, and doing, going through sort of thought process of thinking about what type of leader you want to be.’ (8.78, 34 year, M)

These self-reflections and softer skills also helped in other areas like being able to connect with different team members and build relationships, as stated by one participant: ‘… that stuck with me was more personality profile that was done, and part of that was learning other personality profiles’ (1.90, 43 year, F).

#### Knowledge gaps specific to administrative skills, governance roles and responsibilities

The knowledge gaps were mainly administrative, like managing grievances, complaints, patient safety incidents and difficult staff-related problems. The roles and responsibilities of FP at a management level, including human resource management processes such as progressive discipline, management of absenteeism and finances of a facility, were identified as a clear gap in the training, as indicated by one of participant: ‘Complaint, progressive discipline. Managing absenteeism is something we were never exposed to’ (3.106, 47 year, M). This was echoed across most interviews, especially where the participants were in FP posts, as shown in the following excerpt:

‘I mean, some of the, you know, some of them were just learnt on the job and usually that was in the face of a crisis when it comes to management and that happened quite early on, and then, you know, one also learns and upskills a lot around rules and processes with, with respect to HR.’ (5.183, 53 year, M)

### Resources used by family physicians. FPs to overcome perceived shortcomings of formal training

#### Making use of a community of practice

As explored throughout the study, all the participants discussed various learning points from lived experience versus formal training, and all were able to identify specific knowledge gaps. Depending on their experience in the role, the perceived amount of time available and the importance of the subject matter, many have attempted to close these gaps through the relevant channels to function effectively as leaders within their space.

Participants instinctively learned from and leaned on their local team and other FPs for support during their earlier challenging years. A participant explicitly stated: ‘To be able to learn from, from others, and so, yeah, I mean amongst us and particularly now, we, we have a, a couple of really excellent managers’ (5.218, 53 year, M). A similar concept was stated by another participant: ‘Asking people for help, so, knowing, knowing who my, my Cape Town based resources were’ (9.184, 41 year, M). Some participants were also grateful that they felt well-supported despite being in a reasonably remote facility: ‘We are five family physicians, and we meet monthly, and we also communicate quite often with each other’ (10.220, 39 year, F). Participants often look to other local team members for support and guidance:

‘It’s been a very good learning curve but manageable in the way that there is a lot of support, a lot of senior people that has been … that has (have) experience.’ (8.50, 34 year, M)

#### Governmental circulars and courses

Some participants could not attend specific courses because of time constraints and service delivery expectations. A participant stated that she was challenged in her time management to prioritise attending available training courses:

‘I’ve always put it down in my skills plan that I would like to do management courses, but never actually got there. I think they are always quite time consuming.’ (1.328, 43 year, F)

These participants made use of the DOH circulars and guidelines which were sent out frequently. ‘Having access to resources, like typed up documents and specific things that they … that is available’ (8.221, 34 year, M).

Other participants, with their line managers’ support, could utilise courses through the DOH. ‘Fortunately, the Northern Tygerberg basically allows us to choose courses or subjects we really want to develop ourselves in’ (3.118, 47 year, M) while others sought private courses and diplomas provided externally to enhance their skills. ‘So, subsequently, after family medicine had done like other things, and one of the things I did was the Oliver Tambo Fellowship Program at UCT’ (4.84, 39 year, F). One participant even sought internationally accredited training: ‘… then I’m currently doing a leadership management course through the University of Washington’ (1.350, 43 year, F).

## Discussion

This study used qualitative methodology and framework analysis to describe how previous versions of the postgraduate L&G training module in the UCT MMed curriculum helped prepare newly qualified FPs for the public sector in the Western Cape province of South Africa. The key findings are summarised in four themes: ‘early experiences of qualified FPs’, ‘key leadership qualities’, ‘fit-for-purpose training’ and ‘resources utilised by FPs’.

The results of this study displayed an appreciation of the rich data, given the diverse experiences of the FPs interviewed, the many possible variations of the training module that they were exposed to and how they utilised that information. The first key finding looked at the early experiences of the FP. This theme highlighted the collective lived experience of FPs, with many expressing similar challenges in their jobs. This challenging or ‘teething’ phase post registrar training has been explored in the literature. Morrow et al. undertook a qualitative cross-speciality study in the United Kingdom, looking specifically at the transition from registrar to consultant. This study highlighted similar learning gaps on leadership, service management, people management and exposure to the consultant role.^32^ Acknowledging that this transition period is present across all specialities and even across different resource settings is essential, and this could be an opportunity to provide additional support for the junior consultant.

Another notable finding from this study was the identification of key leadership qualities needed to work within the public sector, as reflected by the participants. Family physicians are not intended to be employed at regional or tertiary hospitals or as clinical managers, as outlined by the latest SAAFP position paper.^10^ More emphasis has been placed on FPs as leaders and custodians within the district health system, especially with South Africa’s health system moving towards National Health Insurance (NHI).^2^ A more recent study exploring the career pathways of new FPs showed that 55.4% of FPs were still in the public sector, with 38.5% of those FPs in leadership positions.^33^ The leadership qualities identified by our participants were aligned with Gilson and Daire’s list of abilities needed to transform health care.^12^ These key qualities, which were similar among all the participants despite various years of experience in their roles, outline two things. Firstly, leadership qualities or abilities are similar in the public sector across leadership roles. Because of this, it is possible that training of senior registrars or junior consultants, in this regard, could be standardised across disciplines. Secondly, it provides evidence that could inform curriculum transformation in post-graduate FM training programmes that better align training and workplace realities.

Following a reflection on their formal training, participants were asked to look at the comprehensiveness of the module and identify strengths and knowledge gaps. A theme from these reflections was the concept of fit-for-purpose training. Reflecting on the importance and relevance of fit-for-purpose training in an editorial from 2011, Burch and Reid explored the many reasons within the SA context for which fit-for-purpose training is necessary.^34^ They discussed the gross inequalities within the health care system, stating that 46% of South Africa’s rural population is served by 12% of the country’s doctors. Furthermore, some of our medical schools have the most significant proportion of emigrating health care professionals worldwide. They had suggested that major curriculum reform, including looking at assessment structures, was needed to address the gross inequities of the SA health system at the time, as well as to seek ways to improve doctor-to-population ratios in public health care facilities and to distribute doctors better to address the health care needs of marginalised communities. The reflections by the participants and the insights from Burch and Reid’s editorial highlight the key feature of fit-for-purpose training, which is essentially that alignment between the curriculum and the future roles and responsibilities of graduates within a specific context. Elaborating further on this, Dudley et al. reviewed the medical undergraduate curriculum and found that health systems, health leadership and management teaching were weak, and essential public health competencies in human rights and health advocacy received little attention. Recent graduates wanted more integrated, practical, problem-based teaching in environments where they would 1 day work and their teachers would be role models for the competencies students were expected to acquire.^31^ Similarly, Taber et al. discussed a fit-for-purpose framework looking at the accreditation of programmes, which appreciates the local contexts of the graduates and the health system in which they will work.^35^ Building on this theme of socially responsive curricular transformation, the findings from our study potentially improve the possibility of SA training programmes becoming more socially just and context specific.

As previously mentioned, participants also reflected on the shortcomings of formal training, and many raised the issue of not being exposed to dealing with daily governance or administrative problems during their registrar time. Participants appreciated the theoretical components of formal training but felt the knowledge gap when utilising these skills when ultimately in the role. This suggests that the formal training could be made fit for purpose by adding a service-learning component, which offers the opportunity for role-modelling clinical leadership. This could include leading morbidity and mortality case meetings, joining finance meetings, sitting on an interview panel or managing a patient safety incident under the guidance of an FP supervisor.

Using a community of practice (COP) to fill knowledge gaps within the clinical leadership role was a key finding. Wenger describes the concept of a COP as being composed of three things: it is a joint enterprise that continually receives input from its members, it functions through mutual engagement from members that bind them together as a society, and this helps them in turn to share and develop a vast number of communal resources. These are created and strengthened by the members around matters important to that society.^36^ This principle of collaborative practice is a core principle of FM and helps strengthen the discipline and the local communities it serves. Leveraging the existing COP to support new consultants or mentor registrars could significantly strengthen the formal training programme.

### Recommendations

The recommendations from this study would be to use the findings in a curriculum review and transformation process, as it pertains to leadership training. The relevant stakeholders could investigate developing a stronger, more dynamic and more relevant module that balances theoretical knowledge with practical skills.

Workplace exposure to the leadership role with different leaders, for example, clinical managers and FPs, could improve graduates’ preparedness to deal with corporate and clinical governance activities and processes. Further research could also be done in other universities to see how they conduct this module and if their graduates have similar feedback. Research can also be undertaken across healthcare disciplines to assess if they run a similar course and then assess the perceived usefulness of the course once graduates are qualified and employed.

Health system leaders could also use these findings to strengthen support systems and resources, especially once FPs are already in management positions. This support package could be extended to all managers and include mandatory introductory courses and short courses on human resources processes that should be encouraged. This could create more confident leaders and managers within district health systems and decrease the time it takes to adjust to their new roles, while improving adherence to governance practices and regulations.

The SAAFP has a mentorship programme (Next5) that aligns with the COP concept and is currently aimed at junior consultants. However, it should also be aimed at final-year registrars so that they can also know their options going forward; this way, the SAAFP would offer holistic support to its members.

### Limitations of the study

The small sample of participants resulted in findings that are not generalisable; however, it offers valuable insights into fit-for-purpose leadership training for FP. Purposive sampling was used; therefore, a wide range of views was not captured. The broad range of years of experience among candidates, coupled with multiple iterations of the L&G module, resulted in a group of study participants with different perspectives and reflections on their version of the module. The deductive approach with framework analysis limits the interpretation to specific themes.

## Conclusion

This qualitative study employed semi-structured interviews to collect data from recently qualified FP consultants regarding their leadership training. The module itself was not viewed as particularly effective in preparing FPs for real-world leadership and governance challenges. The study identified key findings summarised into four themes: ‘early experiences of qualified FPs’, ‘key leadership qualities’, ‘fit-for-purpose training’ and ‘resources utilised by FPs’. These themes helped to illustrate their leadership journeys, emphasising areas of the current training that could be improved. Future research should examine workplace-based leadership training models and the influence of leadership role-modelling and include ongoing assessments of fit-for-purpose training programmes.
